# Crossroads of Cancer and HIV-1: Pathways to a Cure for HIV

**DOI:** 10.3389/fimmu.2019.02267

**Published:** 2019-10-04

**Authors:** Christina Gavegnano, Andrea Savarino, Taofeek Owanikoko, Vincent C. Marconi

**Affiliations:** ^1^Laboratory of Biochemical Pharmacology, Department of Pediatrics, Emory University School of Medicine, Atlanta, GA, United States; ^2^Istituto Superiore di Sanita, Rome, Italy; ^3^Department of Hematology and Medical Oncology, Emory University School of Medicine, Atlanta, GA, United States; ^4^Emory Vaccine Center, Rollins School of Public Health, Emory University School of Medicine, Atlanta, GA, United States; ^5^Atlanta Veterans Affairs Medical Center, Atlanta, GA, United States

**Keywords:** HIV, immunomodulator, inflammation, eradication, latent reservoir HIV infected CD4 T cells, apoptosis of HIV infected CD4 T cells

## Abstract

Recently, a second individual (the “London patient”) with HIV-1 infection and concomitant leukemia was cured of both diseases by a conditioning myeloablative regimen followed by transplantation of stem cells bearing the homozygous CCR5 Δ32 mutation. The substantial risks and cost associated with this procedure render it unfeasible on a large scale. This strategy also indicates that a common pathway toward a cure for both HIV and cancer may exist. Successful approaches to curing both diseases should ideally possess three components, i.e., (1) direct targeting of pathological cells (neoplastic cells in cancer and the HIV-infected reservoir cells), (2) subsequent impediment to reconstitution of the pool of pathological cells and (3) sustained, immunologic control of the disease (both diseases are characterized by detrimental immune hyper-activation that hinders successful establishment of immunity). In this review, we explore medications that are either investigational or FDA-approved anticancer treatments that may be employed to achieve the goal of curing HIV-1. These include: thioredoxin reductase inhibitors (phases 1–3), immune checkpoint inhibitors (phases 1, 3), Jak inhibitors (FDA approved for arthritis and multiple cancer indications, summarized in Table 1). Of note, some of these medications such as arsenic trioxide and Jak inhibitors may also reversibly down regulate CCR5 expression on CD4^+^ T-cells, thus escaping the ethical issues of inducing or transferring mutations in CCR5 that are presently the subject of interest as it relates to HIV-1 cure strategies.

## Introduction

Human Immunodeficiency Virus (HIV-1) is currently well-managed and achieves plasma viral suppression with existing combination antiretroviral therapy (ART). Despite durable virologic suppression, a major barrier to eradication of HIV-1 remains the effective elimination of cells harboring integrated HIV in a latent or low-level replication state, including pharmacological sanctuaries such as the central nervous system (CNS) (1–6). Further, many reports now demonstrate that chronic immune activation and exhaustion transpire in people living with HIV (PLWH) even with well-controlled viremia. These markers include elevated levels of inflammatory and immunomodulatory cytokines including IL-1α/β, TNF-α, IL-6, D-dimer, C reactive protein (CRP), IL-7, IL-15, sCD14, and sCD163. These correlate with increased morbidity and mortality in PLWH (2, 6–16). For example, IL-7/15 drive homeostatic proliferation and IL-15 reactivates HIV-1 from latent stores, thereby expanding the viral reservoir (2, 10, 11). Elevated inflammation is a driver of immune exhaustion (elevated PD-1), which is also associated with disease progression for PLWH (7, 17, 18).

Existing antiretroviral agents do not completely eliminate ongoing inflammation and immune activation for all virologically suppressed individuals, nor do these agents target the latent or persistent viral reservoir (19–21). These limitations have led to the burgeoning exploration of well-defined anti-cancer agents that target key cellular and immunomodulatory pathways.

The interface between cancer and HIV-1 treatment is not new; the first FDA-approved antiviral agent was azidothymidine, an adenosine nucleoside analog originally explored as an anticancer agent to block DNA synthesis given its lack of the 3′ hydroxyl group (22–24). The wealth of FDA-approved agents with well-described mechanisms of action and clinical profiles provide a robust foundation that can be readily leveraged for HIV-1 treatment. This review focuses on key anti-cancer agents that either are FDA-approved or have already begun clinical investigation in PLWH.

## Hematopoietic Stem Cell Transplantation and Cellular Therapy

The concept of disease eradication followed by reconstitution of depleted cell lines using a living, unrelated donor is over 60 years old. The first allogeneic hematopoietic stem cell transplantation (HSCT) was performed in 1957 by E. Donnall Thomas on six individuals with various malignancies (25). Only two patients engrafted after conditioning chemotherapy with radiation, and all died within 100 days. Allogeneic transplants require the bone marrow graft to be derived from a healthy donor, in contrast to an autograft (self) transplantation. The procedure usually requires an initial round of high dose chemotherapy along with total or selective body irradiation to reduce the tumor burden and weaken native immunity prior to transplantation. Thereafter, donor cells are infused, followed by the administration of immunosuppressive agents initially to prevent rejection and later to minimize graft-vs.-host disease (GVHD). After the introduction of histocompatibility matching in the 1980s, disease-free survival dramatically improved as graft rejection and GVHD decreased (26). In the modern era of HSCT, the majority of patients undergoing this procedure have refractory leukemia or multiple myelomas well as other malignancies such as lymphomas (non-Hodgkin's and Hodgkin's), gliomas and neuroblastomas. HSCT has also been an effective therapeutic strategy for non-malignant diseases including autoimmune disorders and sickle cell disease (27, 28). According to the WHO, over 50,000 HSCT are performed worldwide each year for malignant indications, with more than 90 % resulting in a cure (29).

In the early years of the HIV epidemic, HSCT was explored as an option to treat cancer or reconstitute the immune system for people living with HIV (PLWH). For untreated, advanced HIV infection, most patients experienced little clinical benefit and ultimately succumbed to AIDS. Interestingly, several of the allogeneic HSCT recipients had, at necropsy, undetectable levels of HIV from various tissues as late as 10 months after transplant (30). Interestingly, syngeneic transplants recipients remained viremic throughout the post-transplant period indicating the potential value of a graft-vs.-virus effect. As ART became available, the interest in developing HSCT as a strategy to specifically treat HIV disease waned. Outcomes of autologous HSCT for PLWH with lymphoma continued to improve and approached those of HIV negative individuals (31). However, the effectiveness of moderately intensive chemotherapy or HSCT on the HIV reservoir had been minimal due to reinfusion of infected CD4^+^ T cells contaminating autografts, new infection of donor-derived CD4^+^ T cells, and chemotherapy-resistant infected cells.

In 2007, Timothy Brown (the “Berlin patient”) underwent HSCT for relapsed acute myelogenous leukemia (AML). Gero Hütter, the treating physician, identified a donor lacking the CCR5 coreceptor (homozygous for the Δ32 deletion) on CD4^+^ T cells, which is critical for R5-tropic HIV viral entry. ART was discontinued, and subsequently HIV in blood and various tissues were undetectable (32). After more than a decade since transplantation, he remains free from both HIV and AML becoming the first patient ever cured of HIV by this strategy (33). Two additional patients in Boston (who were themselves heterozygous for the Δ32 deletion) underwent HSCT using a reduced-intensity conditioning regimen and CCR5^+^ wild-type donors (34). Unfortunately, both patients experienced viral rebound 12 and 32 weeks after ART cessation despite maintaining undetectable levels while receiving ART until full chimerism was achieved. More recent evidence of HIV remission following HSCT has been documented from a patient in London who is now over 18 months undetectable (35). Numerous protocols are underway to examine this approach in various international settings (30). The Berlin patient prompted new research aimed at knocking–down CCR5 on CD4^+^ T cells using CRISPR/Cas9, zinc-finger nuclease and transcription activator-like effector nuclease genome editing systems (36). There are some concerns around the use of lentiviral transduction resulting in insertional oncogenesis and the potential effect of losing the CCR5 co-receptor for immune function and mortality (37). Furthermore, without fully myeloablative chemotherapy or high efficiency of graft transduction, it is unclear how to best to achieve complete chimerism in the host, and identification of donor matched Δ32 for all PLWH is not possible, given this mutation is a rare mutation in the human population at large (38). Additionally, there are ethical considerations for altering the human genome; modifying CCR5 signal transduction may have implications for specific pathogens and long-term immunity that are incompletely understood.

Despite encouraging evidence, this procedure incurs a significant risk of complications, challenging pharmacological interactions and substantial financial costs. Survival at 1 year is around 60% with the underlying malignancy often described as the cause of death. The major adverse events include infection, liver injury due to veno-oclusive disease, and GVHD. At a median total healthcare cost at 100 days of $289,283 for the myeloablative HSCT and $253,467 for reduced-intensity HSCT (39), allogeneic HSCT is not a viable treatment option for the nearly 37 million PLWH globally. This reason alone has weakened support of HSCT as a viable strategy for HIV cure (40–43). More recently, the use of virally transduced chimeric antigen presenting autologous T-cells (CAR-T) has broadened the potential utility of immune directed anticancer therapy with approval of several agents in this class by the US FDA for the treatment of refractory leukemia and lymphoma (44). While the success of HSCT has been limited to hematologic malignancies, CAR-T has the potential to positively impact the treatment of solid malignancies in the near future. Despite the advancement of this potential intervention for cancer, implementation of CAR-T cells to HIV-positive individuals presents with significant logistic limitations since it requires transplantation, limiting its application to the nearly 37 million PLWH worldwide.

## Checkpoint Inhibitors

There are a number of approved immunotherapeutic agents directed at CTLA-4 (ipilimumab), programmed cell death 1 protein or PD-1 (nivolumab, pembrolizumab, cemiplimab), and PD-L1 (atezolizumab and durvalumab) (45). See Table 1 for summary of indication and route of administration for this class of agents. Each of these monoclonal antibodies present critical pharmacokinetic challenges. For example, they are not orally bioavailable and there is poor tissue delivery of these agents at adequate concentrations to confer efficacy.

**Table 1:** Summary of anti-cancer agents that have been explored for the indication of HIV.

**Agent**	**Target**	**Route of administration**
Nivolumab, pembrolizumab, cemiplimab), and PD-L1 (atezolizumab and durvalumab	PD-1	Monoclonal antibody; infusion
Auranofin	Thioredoxin reductase	Oral
Arsenic trioxide	Thioredoxin reductase	Intravenous
Ruxolitinib	Jak 1/2	Oral
Baricitinib	Jak 1/2	Oral

Immunologic dysfunction associated with HIV infection and persistence, including T cell exhaustion, is related to overexpression of checkpoint molecules including CTLA-4, PD-1, LAG-3, and TIM-3. This overexpression is a major contributor of the viral reservoir in ART-suppressed, HIV-positive patients and non-human primates (46–48). Recently, a report from Fromentin et al. demonstrated that PD-1 blockade potentiates HIV latency reversal *ex vivo* in CD4^+^ T cells from ART-suppressed individuals (49), further underscoring the role of PD-1 in HIV-1 latency, reversal, and overall reactivation.

Clinical trials are already underway (NCT02408861, NCT03354936) or have been completed to test checkpoint blockade. In a previous case report, ipilimumab was given to a HIV positive patient with melanoma. This patient experienced an increase in CD4^+^ T cell quantity, T cell activation and cell-associated unspliced HIV RNA with a subsequent decline in plasma HIV RNA (50). Moreover, a HIV-positive patient with lung cancer was given nivolumab with a subsequent reactivation of latently-infected T cells (51). Significant adverse effects have been reported when using these agents in cancer; as these molecules are involved in antigen self-tolerance, disruption can lead to autoimmune or inflammatory side-effects, reactivation of underlying autoimmune conditions, or new autoimmune conditions such as type 1 diabetes mellitus (52). Several case reports have described colitis, skin toxicities, endocrinopathies, pneumonitis, and hepatitis (53, 54). Finally the substantial cost of these agents necessitates a careful consideration of which patients and populations would be ideal candidates for this class of drug (55). Together, these significant safety limitations coupled with cost of treatment, likely preclude their development for the indication of HIV-1 cure.

## Thioredoxin Reductase Inhibitors

Thioredoxin reductase (TrxR) is a key suppressor of oxidative stress and regulates cell death and differentiation. It is a selenoprotein which reduces the oxidized from of thioredoxin (Trx), turning this protein into its active reducing form, thus maintaining the functional levels of one of the main cellular antioxidants (56). The presence of a selenocysteine in the active center of TrxR renders it sensitive to inhibition by a number of metal and metalloid ions, which directly bind the selenium ion of selenocysteine thus blocking the active center of the protein (57).

Auranofin is the only gold salt which is orally available and FDA-approved, see Table 1 for summary of indication and route of administration (58, 59), although it is rarely prescribed in the modern era due to toxicities, and development of other more specific, safe and well tolerated agents. Auranofin was developed for RA treatment in the 1970s, but, at that time, the mechanisms behind its effects on the immune system were largely unknown (58). It was known, however, that the compound inhibited lymphocyte proliferation (60), and, in this light, its anticancer potential soon became apparent (61). A recent human clinical trial with five HIV-positive individuals was conducted (NCT02961829) (62). The findings demonstrate that no severe adverse events were reported for the duration of the study, apart from a decline in total CD4 T cells at week 8 and week 12. A sample size of five individuals per group, statistical analysis to confidently perform appropriate statistical tests to determine significance of findings cannot be performed; nonetheless, the trial demonstrates that auranofin may be safely tolerated in HIV-positive individuals; further studies are needed to better understand the impact of this agent on the viral reservoir.

To date, auranofin has been largely replaced by modern-era anti-cancer agents that demonstrate a significant improvement in safety and specificity profiles. Nonetheless, the ability of this agent to block activation based events that drive immune activation add to a better understanding of links between inflammatory events and HIV persistence.

## Arsenic Trioxide (ATO)

Early reports demonstrated that ATO potently suppressed lymphocytic proliferation in acute promyelocytic leukemia (APL) (63), however the fact that it blocks T cell proliferation provides serious concern for application toward PLWH, given CD4 T cell loss is a major hallmark of disease pathology in this population. A case-report study demonstrated that oral arsenic trioxide–based maintenance regimens conferred complete remission of APL in a 10-year follow up study, underscoring that agent can be tolerated in this cohort to achieve remission (64). APL requires a 15:17 chromosome translocation and chimerization of the retinoic acid-RAR-α and the promyelocytic leukemia protein (PML). PML is a primary constituent of the nuclear bodies, a molecular “hub” attracting chromatin-modifying enzymes and transcription factors regulating cell death and proliferation and, interestingly, HIV-1 transcription (65). A combination of the RAR-α ligand all-trans retinoic acid and ATO, found to be a PML ligand (65), has become an effective, FDA-approved treatment for APL, inducing stable remission of the disease (66). See Table 1 for summary of indication and route of administration. Despite this approval, arsenic-based compounds are considered to be toxic, although the benefits to patients with cancer may outweigh the risks. It remains to be seen whether this risk-benefit ratio will be similar for PLWH. More recently in the past decade, the inhibitory effects of ATO on TrxR were discovered (67). ATO was thus tested, alone or in combination with other drugs in a wide variety of cancers without the 15:17 chromosome translocation including solid malignancies such as melanoma and small cell lung cancer (68, 69) and was approved as a treatment for hepatocellular carcinoma in China (70).

Currently, ATO could provide insight into mechanisms for cure-based work for several reasons: First, ATO has been reported to demonstrate efficacy in the traditional “shock and kill” strategy, with a mechanism that is related to TxR inhibition (65, 71), which implies a relationship between TxR inhibition and viral reactivation. Additionally, ATO reduces the susceptibility of subsequent HIV infection down-regulating CCR5 expression on CD4^+^ T-cells without the need of a bone marrow transplantation. A recent report also stated that ATO (72) conferred a delay in viral rebound for two SIV-infected macaques (out of four total animals in the study), with doses similar to those administered to humans with APL. A larger sample size to determine the impact of these agents in macaques is warranted, although concern for overall T cell loss with an anti-leukemic agent in PLWH will require a thorough evaluation of the risk/benefit ratio. CCR5 down-regulation is the likely result of the pro-differentiating effects of ATO in lymphocytes: similarly to auranofin, ATO induces CD27 down-regulation, thus limiting their potential to become activated (73). A Phase 1 human study (20 total participants randomized to control or treatment groups) to examine this agent is currently recruiting in China, which may provide critical insight into the safety and efficacy of this agent in PLWH (https://clinicaltrials.gov/ct2/show/NCT03980665). Together these mechanisms provide insight into control of the viral reservoir, although direct clinical application of ATO to HIV-positive individuals is uncertain at present. Weighing the risk/benefit ratio for an agent that may block pro-HIV events, vs. its clinical safety profile must be carefully considered with agents, especially those that are not prescribed currently due to their safety profiles.

## Jak Inhibitors

The Janus activating kinase signal transducer and activator of transcription (Jak-STAT) pathway is activated within 2 h of HIV-1 envelope gp120 binding to CD4, in both primary T-cells and macrophages, in a co-receptor independent manner (74). Downstream activation results in Jak activation, subsequent STAT phosphorylation, and extracellular production of pro-inflammatory and cytokines that are key drivers of HIV persistence, disease progression, reservoir magnitude, and decreased CD4 T cell counts (7, 75–78).

Jak 1/2 inhibitors including ruxolitinib (Jakafi; Jakafi.com), and baricitinib (olumiant; olumiant.com) are FDA-approved for myelofibrosis or polycytemia vera (ruxolitinib), and rheumatoid arthritis (baricitinib), respectively. Baricitinib is FDA-approved for long-term use, including in children, rendering its safety profile favorable for consideration in PLWH. See Table 1 for summary of indication and oral administration for jak inhibitors. Jak 1/2 blockade represents an attractive cellular target because Jak 3 but not Jak 1/2 blockade induces natural killer cell depletion and systemic side effects that can promote immunosuppression (79–82). Ruxolitinib has demonstrated potent inhibition of reservoir establishment, maintenance and expansion in primary T cells and macrophages *in vitro* and *ex vivo* (7), and demonstrated reduction in immune activation markers associated with HIV-1 persistence including CCR5, HLA-DR, CD38, CD25, Ki67, and PD-1. Further, Jak inhibitors significantly reduce Bcl-2 expression in non-dividing p24^+^ primary T cells *ex vivo*, thereby offering the potential to reduce the lifespan of reservoir cells by down-regulating a key marker that controls lifespan of cells (7, 14, 78, 83, 84). These data provided the foundation for a recently completed multi-site Phase 2a AIDS Clinical Trial Group (ACTG)-funded study (https://www.clinicaltrials.gov/NCT02475655. It was recently reported that ruxolitinib was safe and well-tolerated in a highly-selected cohort of PLWH on suppressive ART (85). The ruxolitinib arm demonstrated a trend in reduction of IL-6, and a statistically significant decrease in sCD14 (85), coupled with an increase in circulating T cells through undefined mechanisms. Data are forthcoming about the impact of this agent on viral reservoirs, and key markers of viral persistence.

Additional work has begun to explore another Jak 1/2 inhibitor, baricitinib for HIV (86). Baricitinib is an FDA approved once-daily dosed, orally bioavailable inhibitor that is renally cleared, approved for long-term use in children (primary indication rheumatoid arthritis, and under investigation for various inflammatory or malignant indications). A recently published study demonstrated that baricitinib reverses HIV associated neurocognitive disorders in a severe combined immunodeficiency (SCID) mouse model and reservoir seeding *in vitro* (86). Importantly, baricitinib was shown to significantly reduce activated phagocytic cells from the periphery that recruit to the CNS during HIV infection, highlighting the link between blockade in the periphery and potential application to CNS infection with HIV. Together, these data provide a rationale for future studies of Jak inhibitors in PLWH who have residual inflammation or immune dysfunction despite long-term suppressive ART.

## Previous Work Paves a Road Toward a Bright Future

Many early studies exploiting anti-cancer agents were based on the “shock and kill” concept (87, 88). These agents, notably panobinostat (primary indication: multiple myeloma) and vorinostat (primary indication: cutaneous T cell lymphoma) failed due to toxicity and lack of efficacy (89). Other modalities targeting key pathways reactivating the latent virus, including protein kinase C (These markers include elevated levels) agonists and toll-like receptor TLR agonists, are being explored, however to date no agent has demonstrated durable reduction in the latent reservoir (90). These agents also may promote systemic immune activation since they are reactivation agents, which could have fuel HIV persistence, representing a major limitation associated with these approaches. Nonetheless, these studies provide a better understanding of potential application of anti-cancer agents and the effect of the shock and kill approach on the HIV reservoir (i.e., reactivation of the latent virus followed by elimination of the infected cell).

## Common Strategies for Curing HIV and Cancer

Curing cancer has a major mechanistic hallmark of stopping proliferation of malignant cells and/or inducing cell death, while maintaining immune function and reducing toxicity of uninfected cells. HIV-1 eradication strategies are now beginning to adopt this paradigm to target and eliminate only HIV-infected cells. This archetype represents the beginning of a new horizon, where better understanding of the complex and delicate interplay between cellular signaling, inflammation, autocrine and paracrine events, and the impact of these events both locally and across organ compartments. The field of HIV has been able to move forward with much greater speed and knowledge due to the wealth of information collected from anti-cancer approaches spanning diverse mechanisms of action. The data collected to date have provided insight into some approaches that are not viable, while simultaneously providing insight into why they may have failed, providing a potential pathway to re-evaluate these mechanisms with different agents. Other approaches have provided promising preliminary data in humans and will require further rigorous evaluation in PLWH. Careful consideration for agents that are safe, specific, and potent that can be translated for large-scale use in PLWH, including children, must be considered. Further, the bioavailability of the agent, its pharmacokinetic profile, and ability to be administered without drug-drug interactions to PLWH who are receiving ART are critical components to repurpose oncology chemotherapeutic agent for use in HIV infection. The data generated to date will facilitate better understanding of the potential impact of these agents on the viral reservoir and end-organ disease, and provide great potential to identify a candidate agent that can lead to a functional HIV cure.

## Conclusions

Targeting and eliminating HIV-infected cells without conferring toxicity to uninfected cells systemically remains a critical key to HIV eradication. The information gained from oncology and its rapidly advancing target library will undoubtedly continue to guide eradication strategies for HIV-1. Data learned from previous work provides hope that eradication of HIV-1 is possible, when guided by the lighthouse of cellular-factor targeted agents and anti-cancer therapies.

## Author Contributions

CG was the primary author and also edited all other contributing portions into the doucment. AS provided sections for thioreductase inhibitors, auronafin, and historical perspectives. TO provided sections for anti-cancer agents and crosstalk to background and clinical application in cancer. VM was the senior author and wrote historical sections, edited document, and guided CG toward completion and structure of the manuscript.

### Conflict of Interest

The authors declare that the research was conducted in the absence of any commercial or financial relationships that could be construed as a potential conflict of interest.

## Abbreviations

HIV: Human Immunodeficiency Virus
CRP: C Reactive Protein
PLWH: People Living with HIV
ART: antiretroviral therapy
HSCT: hematopoietic stem cell transplantation
GVHD: graft-versus-host disease
TrxR: Thioredoxin reductase
ATO: Arsenic trioxide
PML: promyelocytic leukemia protein
Jak-STAT: Janus activating kinase signal transducer and activator of transcription
PKC: protein kinase C
TLR: toll-like receptor.
